# Modulating immune cells within pancreatic ductal adenocarcinoma via nanomedicine

**DOI:** 10.1042/EBC20243001

**Published:** 2025-05-26

**Authors:** Junyi Lin, Ying Li, Jingjing Sun

**Affiliations:** 1 Department of Pharmaceutical Sciences, College of Pharmacy, University of Nebraska Medical Center, Omaha, Nebraska 68106, U.S.A.; 2 Fred & Pamela Buffett Cancer Center, University of Nebraska Medical Center, Omaha, Nebraska 68106, U.S.A.

**Keywords:** immune cell, immunology, nanocarriers, pancreatic ductal adenocarcinoma, tumor microenvironment

## Abstract

Pancreatic ductal adenocarcinoma (PDAC) is an aggressive malignancy characterized by a dense extracellular matrix (ECM) and a uniquely immunosuppressive tumor microenvironment (TME), which together form a formidable barrier that hinders deep drug penetration, limiting the efficacy of conventional therapies and leading to poor patient outcomes. Nanocarrier technology emerges as a promising strategy to improve treatment efficacy in PDAC. Nanocarriers can not only improve drug penetration through their adjustable physicochemical properties but also effectively regulate immune cell function in pancreatic cancer TME and promote anti-tumor immune response. This mini-review discusses the effects of nanocarriers on the immune microenvironment of PDAC, analyzing their mechanisms in modulating immune cells, overcoming ECM barriers, and reshaping the TME.

## Introduction

Pancreatic ductal adenocarcinoma (PDAC) is one of the most aggressive malignancies worldwide, characterized by late diagnosis, rapid progression, and resistance to conventional therapies [1–5]. In the United States, PDAC remains a major health burden, with a five-year survival rate of just 11% [6]. In Asia and Europe, the incidence rates are increasing. This situation has spurred extensive research efforts on understanding the role of the tumor microenvironment (TME) in PDAC progression and developing innovative therapeutic strategies [7, 8]. A major challenge in treating PDAC lies in its complex TME, which consists of a dense extracellular matrix (ECM) and extensive desmoplasia (overproduction of fibrotic tissue due to excessive stromal activation) [9]. This not only restricts drug delivery but also physically limits immune cell infiltration, leading to a highly immunosuppressive TME. In addition, various cytokines and chemokines are secreted in the TME, which promotes immune evasion, making PDAC particularly resistant to immunotherapies compared with other tumor types. Therefore, understanding and overcoming the barriers posed by the PDAC TME has become a critical area of research.

The role of immune cells within the TME, such as tumor-infiltrating T cells, natural killer (NK) cells, dendritic cells (DCs), and tumor-associated macrophages (TAMs), is central to determining the outcome of the immune response. However, in PDAC, these immune cells often exhibit impaired function or are polarized toward immunosuppressive phenotypes due to the influence of cytokines, chemokines, and other soluble factors in the TME. Modulating these immune cells to restore their anti-tumor activity has emerged as a promising therapeutic strategy.

Nanomedicine has shown great potential in addressing the unique challenges of the PDAC TME. With their tunable size, surface properties, and ability to deliver therapeutic agents in a controlled and targeted manner, nanocarriers offer a powerful tool for enhancing drug delivery, modulating immune responses, and reprogramming immune cells within the TME. This mini-review explores recent research on using nanomedicine to modulate immune cells within the PDAC TME. We first discuss the key characteristics of the TME in PDAC and the challenges it poses for immunotherapy. Following this, we highlight recent advancements in targeting specific immune cell populations—such as tumor-infiltrating T cells, NK cells, DCs, myeloid-derived suppressor cell (MDSC), and TAMs—using nanocarriers. By providing an overview of these emerging strategies, we aim to clarify why nanomedicine holds significant promise in the modulation of immune cells for the treatment of PDAC.

### Characteristics of pancreatic cancer TME

PDAC presents a uniquely challenging TME compared with other solid tumors, largely due to its highly fibrotic and dense ECM. The fibrotic reaction, also known as desmoplasia, begins to form early around the lesions of pancreatic intraepithelial neoplasia (PanIN). In advanced stages, the ECM can account for up to 90% of the tumor volume. Pancreatic stellate cells (PSCs) are resident cells in the pancreas that play a crucial role in ECM production [10]. Cytokines and growth factors, including insulin-like growth factor, TGF-β, and tumor necrosis factor-α, further stimulate PSC proliferation and exacerbate ECM synthesis, leading to abnormal fibrosis and ECM accumulation [11]. The ECM of PDAC is rich in components such as collagen, proteoglycans, hyaluronic acid (HA), and fibronectin, forming a dense and impermeable barrier [12,13]. This fibrous matrix not only physically obstructs drug penetration and immune cell infiltration but also increases interstitial pressure, reducing blood vessel density and causing low vascular permeability within the tumor [14].

**Figure 1:**
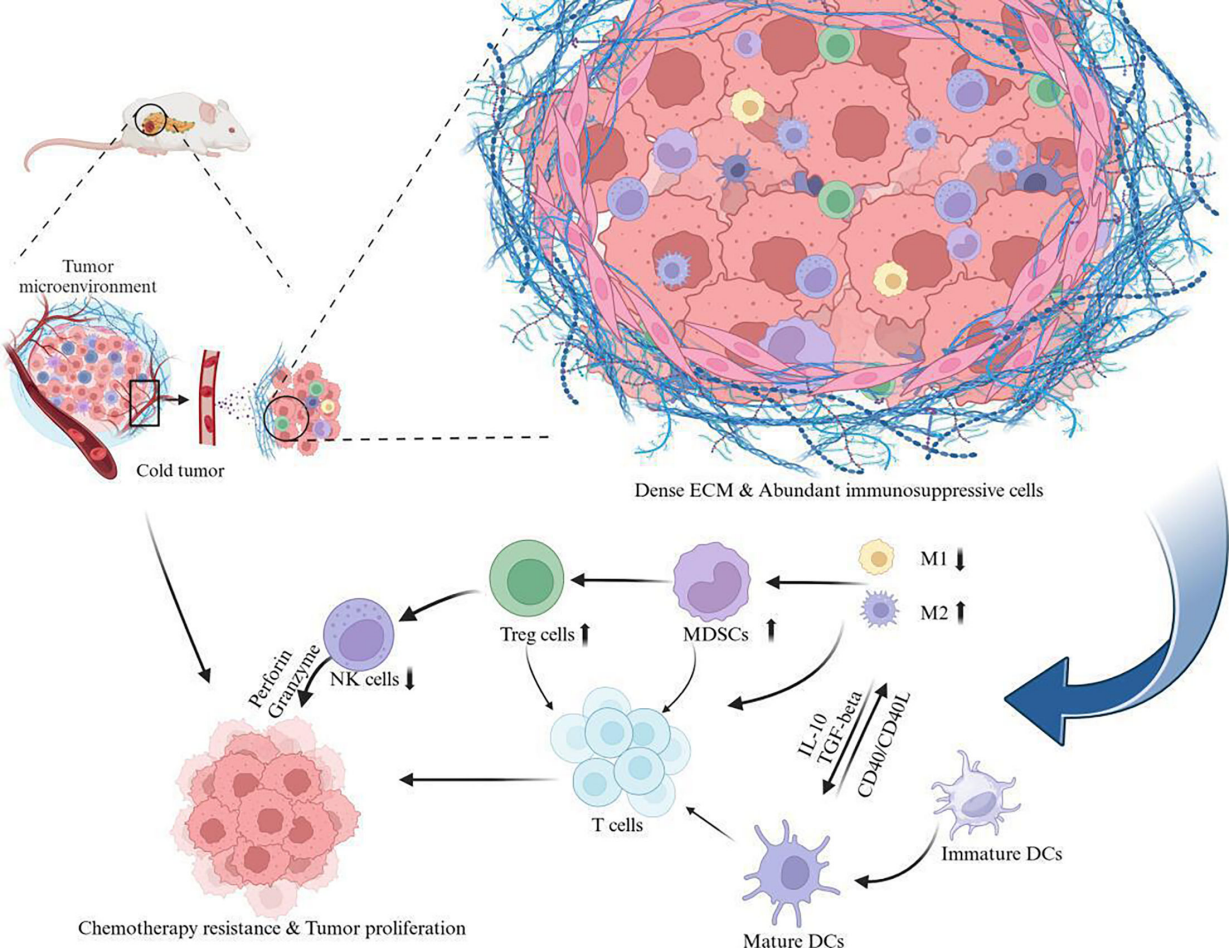
Schematic representation of the uniquely challenging TME characteristic of PDAC. The ECM and abundant immunosuppressive cells, such as Tregs and MDSCs, hinder immune infiltration and anti-tumor activity. Immune cells are dysregulated, with increased MDSCs, Tregs, and M2 macrophages, while cytotoxic T cells and NK cells are suppressed. These features promote chemotherapy resistance, tumor proliferation, and immune evasion, sustaining the cold tumor phenotype. MDSCs, myeloid-derived suppressor cells; NK, natural killer; PDAC, pancreatic ductal adenocarcinoma; TME, tumor microenvironment; Tregs, regulatory T.

In addition to creating physical barrier that restricts immune cell infiltration, components of the TME and tumor cells secrete various immunosuppressive molecules, such as IL-10, TGF-β, and vascular endothelial growth factor (VEGF). These factors promote the recruitment and maintenance of immunosuppressive cells such as TAMs, MDSCs, and Tregs, while simultaneously suppressing the activity of effector immune cells (Figure 1). TGF-β, in particular, plays a central role in inhibiting NK cells and T cell activity, further reinforcing immunosuppression within the TME [15].

Due to the high interstitial pressure and poor vascularization resulting from the dense ECM, the PDAC TME is often hypoxic (low oxygen levels). Hypoxia leads to the stabilization of hypoxia-inducible factors, which promote the expression of immunosuppressive cytokines and the recruitment of immunosuppressive cells. Tumor cells adapt to these hypoxic conditions by undergoing metabolic reprogramming, leading to increased nutrient competition between tumor cells and immune cells. This competition deprives anti-tumor immune cells, such as T cells, of essential nutrients, impairing their function and further enhancing immune evasion within the tumor.

Together, the dense ECM, activated PSCs, immunosuppressive factors, and hypoxic environment create a formidable barrier in PDAC, limiting the efficacy of conventional therapies and immunotherapies. The development of new strategies to overcome these barriers is critical for improving outcomes in PDAC.

### Nanomedicine to overcome the ECM barrier

Ultrasmall nanoparticles have been developed to effectively penetrate the stroma-rich environment of pancreatic tumors. Unlike larger particles, these smaller nanoparticles can better reach the tumor core, bypassing the dense ECM that typically restricts drug delivery [16]. These nanocarriers can be used to deliver chemotherapeutics and immunotherapeutics deeply into the tumor core for better therapy. Nanoparticles can be designed to target stromal cells, such as cancer-associated fibroblasts, and aim to reduce ECM production, ultimately decreasing the physical barrier to therapeutic agents [17]. To overcome both ECM barrier and immunosuppressive microenvironment, Wang et al. [18] developed a small sized polyamino acid-based nanodrug incorporating the PSC activation inhibitor calcipotriol and anti-CXCL12 siRNA. This dual-delivery system works to penetrate pancreatic tumors, inactivate PSCs, and down-regulate CXCL12. This remodeled the TME by decreasing the ECM and immunosuppressive T cells, enhancing cytotoxic T cell infiltration and boosting the efficacy of immune checkpoint blockade (ICB) therapy in ‘cold’ pancreatic tumors. Liu et al. [19] developed doxorubicin (DOX)-supported silica nanocarriers (DOX@HMSPHs) functionalized with hyaluronidase to degrade HA in the ECM, enhancing tumor penetration and inducing immunogenic cell death (ICD). The DOX@HMSPHs release DOX in acidic conditions, triggering ICD, promoting DC antigen presentation, and facilitating T cell activation.

### Nanomedicine strategies to modulate tumor-infiltrating T cells

Tumor-infiltrating T cells are key immune components in the PDAC TME, comprising CD8^+^ effector T cells, CD4^+^ helper T cells, and regulatory T cells (Tregs) [20]. Among them, CD8^+^ T cells serve as primary cytotoxic agents against malignant tumor cells [21]. However, PDAC is often classified as a ‘cold tumor’, characterized by limited T cell infiltration and functional exhaustion of T cells due to the highly immunosuppressive TME [22]. In this hostile environment, factors such as tumor-secreted TGF-β and PD-L1 expression hinder the function of tumor-infiltrating T cells, reducing their effectiveness against cancer cells [23,24]. CD8^+^ T cells, in particular, frequently exhibit an ‘exhausted’ phenotype, marked by the overexpression of inhibitory receptors such as PD-1 and CTLA-4, which impairs their cytotoxic activity [25,26].

**Figure 2:**
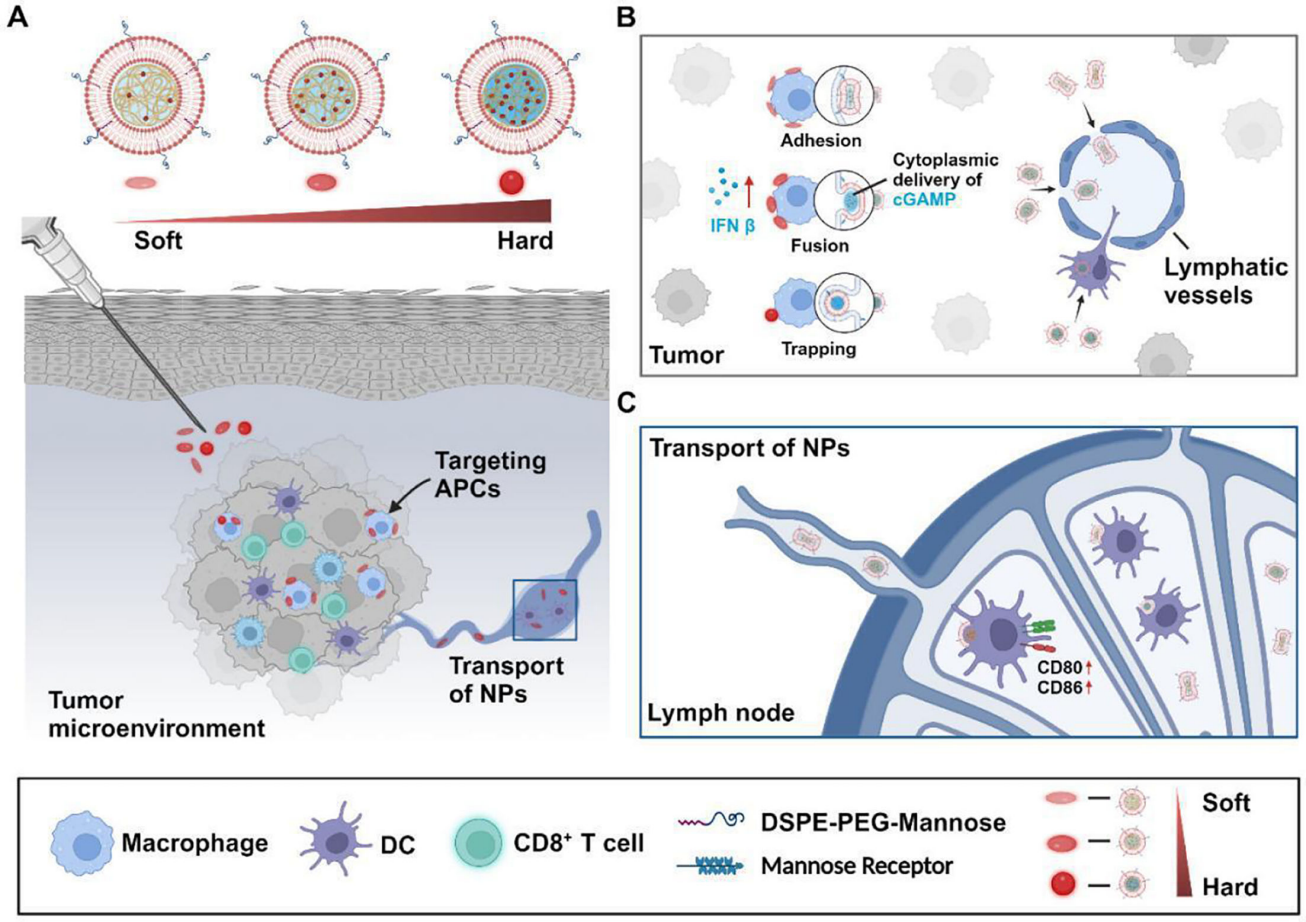
Schematic representation of how the elasticity of Lipo-NPs influences core delivery and immune activation. (**A**) Depiction of local tumor administration for three types of Lipo-NPs. (**B**) Comparison of APC internalization and lymphatic vessel trafficking among the three Lipo-NPs with varying elastic properties. (**C**) The elasticity-dependent transport of Lipo-NPs to tumor-draining lymph nodes (tdLNs) following local tumor administration. Adapted with permission from Zhu et al. [27]. Copyright 2024 American Chemical Society. APC, antigen-presenting cell; Lipo-NPs, liposome nanoparticles.

To overcome these challenges, various nanomedicine strategies have been developed to modulate T cells and enhance their anti-tumor efficacy in PDAC. One promising approach by Zhu et al. [27] involves a TME-activable prodrug nanoparticle to enhance tumor penetration and achieve synergistic anti-tumor effects with chemotherapy and ICB. The nanoparticle contains a PD-L1 antagonist (DPPA) conjugated to a DOX prodrug, along with PEGylated DOX, which dissociates into small nanoparticles (<30 nm) in the TME, releasing DPPA. This co-delivery system improves tumor accumulation and penetration via transcytosis, directly killing tumor cells, promoting cytotoxic T cell infiltration, reducing Tregs, and inducing long-term immune memory to prevent recurrence and metastasis, and shows promise for chemoimmunotherapy in solid tumors. This TME-activable nanoparticle offers a potential platform for enhancing immunochemotherapy in solid tumors. Another approach focuses on modulating the physical properties of nanoparticles to improve immune interactions. Yuan et al. [28] conducted an extensive study on liposome nanoparticles (Lipo-NPs) with varying elastic properties, focusing on their interactions with immune cells and their transport mechanisms from tumors to tumor-draining lymph nodes (tdLNs) (Figure 2). They prepared Lipo-NPs with soft, moderate, and hard elasticity and observed distinct behaviors in immune cell interactions. Soft Lipo-NPs displayed an affinity for cell membranes, while moderate-elastic Lipo-NPs facilitated cargo delivery to macrophages through membrane fusion. In contrast, hard Lipo-NPs entered macrophages via a traditional cellular uptake pathway. Among the tested formulations, moderate-elastic Lipo-NPs loaded with the cGAMP agonist were particularly effective. These nanoparticles promoted significant tumor-infiltrating lymphocyte infiltration by activating the interferon gene stimulator (STING) pathway and enhancing transport to tdLNs, which led to substantial anti-tumor effects and prolonged survival in a mouse melanoma model. This study highlights the potential synergies of moderately elastic Lipo-NPs with ICB therapy in preventing tumor immune escape. These findings offer valuable insights for developing immune-targeted delivery systems, especially in designing tdLN-targeted vaccines aimed at eradicating metastasis within tdLNs. In addition to these approaches, combining Toll-like receptor (TLR) 7/8 ligands with radiotherapy has shown promising results. Zhang et al. [29] developed a linker-based strategy to control the activation of TLR7/8 agonists in PPS nanoparticles (PPS NPs). By attaching agonists to PPS NPs using different linkers, they enhanced therapeutic effects while reducing systemic toxicity. The alkyl linker selectively prolonged DC activation, limiting excessive immune responses and minimizing toxicity. This approach demonstrated strong anti-tumor effects and increased tumor-specific CD8^+^ T cells.

Since Tregs contribute to the immunosuppressive environment in PDAC, reducing their presence has been a focus of several nanomedicine strategies [30–33]. For instance, Sun et al. [34] developed gemcitabine (GEM) conjugated polymer (PGEM) that co-delivered the indoleamine 2,3-dioxygenase 1 inhibitor NLG919 and chemotherapy agent paclitaxel (PTX). This combination reduced Tregs and increased CD4^+^ and CD8^+^ T cells expressing IFN-γ, enhancing the anti-tumor immune response and inhibiting tumor growth in PDAC models. This co-delivery strategy via nanoparticles offers an effective method for enhancing tumor penetration and improving immunochemotherapy outcomes.

### Restoring NK cells function in PDAC through nanomedicine

NK cells are essential effector cells within the innate immune system, playing a crucial role in anti-tumor and antiviral responses. NK cells can recognize and directly eliminate tumor cells without prior activation. However, the functionality of NK cells is often impaired in tumors like pancreatic cancer due to immune suppressive factors and cellular interactions within the TME, which diminish their cytotoxic capacity, allowing tumor cells to evade immune surveillance [35].

Li et al. [36] designed a liposome conjugated with the tumor-homing peptide iRGD (c(CRGDKGPDC)), enabling enhanced tumor penetration and cell uptake for co-delivery of STING agonists and STAT3 inhibitors. These nanoparticles regulate the STING/STAT3 signaling axis and effectively inhibit tumor proliferation and survival. Treatment of the nanoparticles significantly increased the activation of NK cells and CD8^+^ T cells in tumors, resulting in robust innate and adaptive immune responses. Deng et al. [37] developed engineered nanogels coated with Panc02 cell membranes to inhibit tumor-derived prostaglandin E2 (PGE2) and enhance NK cell activation, promoting non-antigen-specific tumor elimination. The nanogels release acetaminophen on demand to reduce PGE2 secretion, while activated NK cells recruit immature DCs and stimulate their maturation, leading to antigen-specific CD8^+^ T cell proliferation. The nanogels demonstrated significant therapeutic effects against Panc02 pancreatic tumor growth and recurrence, particularly when combined with PD-L1 checkpoint blockade therapy, offering a novel strategy to improve immunotherapy in low-immunogenic tumors.

Yang et al. [38] demonstrate how a programmed nanoremodeler (DAS@P/H/pp) restores NK cell function in PDAC. Under the acidic TME, the nanocarrier undergoes charge reversal, triggering the release of hyaluronidase, which degrades the ECM. This process enhances the recruitment and infiltration of NK cells into deep tumor tissues, while promoting the delivery of immunoregulatory molecules and chemotherapy drugs. In a mouse model of pancreatic cancer, this nanomediated strategy significantly boosted the tumor-killing capabilities of NK92 cells. Near-infrared-II fluorescence imaging was used to monitor the treatment efficacy in real time. This approach highlights the potential of nanomedicine in overcoming the immunosuppressive microenvironment of PDAC and restoring anti-tumor function of NK cell.

### Nanomedicine-based restoration of DCs function

DCs are essential components of antigen-presenting cells (APCs) that play a pivotal role in the TME of PDAC [31]. Mature DCs present tumor antigens to CD8^+^ cytotoxic T cells via MHC-I and to CD4^+^ helper T cells via MHC-II, inducing a specific anti-tumor immune response [39]. However, their function is often compromised in the highly immunosuppressive TME of PDAC [32,33]. Factors such as TGF-β, IL-10, and VEGF, released by tumor cells, inhibit DC maturation, impairing their antigen-presenting capability [40–43]. These immature DCs lack the ability to effectively activate T cells and may instead promote immune evasion and tumor progression [44].

Recent studies [45] have shown that manganese (Mn)-based MOF nanoparticles grafted with Peg alone exhibit potent therapeutic effects against lethal pancreatic tumors, demonstrating significant cancer cell-killing activity both *in vitro* with pancreatic cancer cells and *in vivo* in orthotopic pancreatic tumor models. Preliminary analysis suggests that Mn ions released from Mn-MOF may enhance the percentage of DCs within the TME, activating them to up-regulate the expression of co-stimulatory molecules CD80^+^, CD83^+^, and CD86^+^ in tumor tissues. Future efforts to precisely control metal ion release and improve tumor-targeting capability are expected to further enhance the potential of Mn-MOF in cancer immunotherapy.

To further boost DC function, Lorkowski et al. [46] developed a highly potent immune-stimulating nanoparticle carrying dual agonists targeting STING and TLR4 pathways. The study demonstrates that adjusting the ratio of two agonists in immune NPs achieves functional synergy, resulting in an 11-fold increase in IFNβ production compared with single-agonist variants. In an *in situ* Panc02 mouse model of PDAC, systemic administration allowed immune NPs to localize predominantly in the APC-rich perivascular area of the tumor, where over 56% of DCs absorbed the NPs. This led to a significant expansion of APCs, with an 11.5-fold increase in lymphocyte infiltration in DCs and throughout pancreatic tumors compared with untreated controls.

### Targeting MDSCs in PDAC with nanomedicine

MDSCs are a heterogeneous population of bone marrow-derived cells that exhibit potent immunosuppressive functions within the TME. In pancreatic cancer patients, MDSC numbers are significantly elevated, and their presence is strongly associated with tumor progression and resistance to therapy [47]. MDSCs inhibit the activity of both T cells and NK cell activity through the production of molecules such as nitric oxide synthase, arginase, and reactive oxygen species (ROS) [48]. Gu et al. [41] demonstrated that MDSCs in pancreatic cancer promote the production of ROS, leading to T cell apoptosis and functional impairment. In addition, MDSCs promote Treg recruitment and release IL-10 and TGF-β, further contributing to the immunosuppressive environment [42].

Various nanomedicine strategies target MDSCs to improve therapeutic outcomes. For example, LY364947, a TGF-β inhibitor, encapsulated in mesoporous silica nanoparticles, reduced MDSC-induced immunosuppression, enhancing GEM delivery and decreasing tumor growth in pancreatic cancer models [43].

Lu et al. [49]. introduced low molecular weight heparin-based nanoparticles (PLT/PTX NPs) that inhibited MDSC recruitment by blocking P-selectin/PSGL-1 interactions, thereby enhancing the immune microenvironment and reducing spontaneous metastasis in pancreatic cancer.

**Figure 3:**
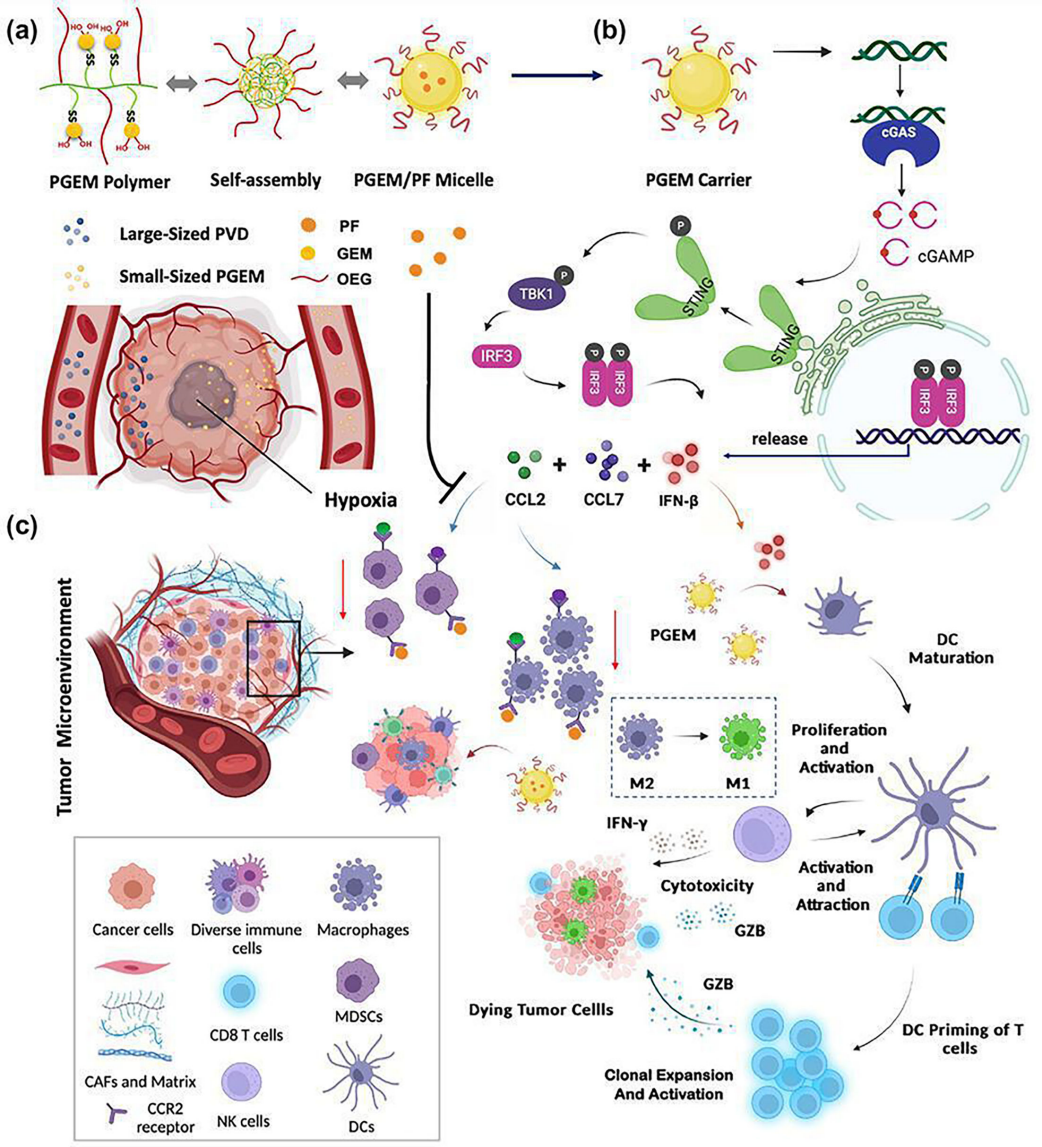
PGEM/PF micelles demonstrate strong anti-tumor effects by reshaping the TME and enhancing STING pathway activation. (**a**) Small-sized PGEM/PF micelles were prepared to facilitate efficient penetration into PDAC solid tumors following systemic administration. (**b**) PGEM effectively activated the STING pathway, leading to the phosphorylation of TBK1 and IRF3, which in turn promoted the production of IFNβ, as well as chemokines CCL2 and CCL7. (**c**) The elevated IFNβ levels induced by PGEM enhanced anti-tumor immunity by facilitating dendritic cell (DC) maturation and stimulating both innate (NK cells) and adaptive (CD8^+^ T cells) immune responses. Simultaneously, CCL2 and CCL7 contributed to recruiting tumor-associated macrophages (TAMs) and myeloid-derived suppressor cells (MDSCs) via CCR2 signaling. Additionally, PF released from PGEM/PF micelles further bolstered anti-tumor immunity by mitigating the immunosuppressive TME through its interplay with the STING pathway. Adapted with permission[50]. Copyright 2023, Mater Today. NK, natural killer; PDAC, pancreatic ductal adenocarcinoma; PGEM, gemcitabine-conjugated polymer.

Wan et al. [50] developed a PGEM micelle system that shows excellent permeability in pancreatic tumor models and acts as a ‘STING agonist’. PGEM activates STING signaling in both tumor cells and DCs, enhancing NK and T cell responses. However, in tumor cells, PGEM-induced STING activation also promotes chemokines CCL2 and CCL7, recruiting immunosuppressive TAMs and MDSCs. To counter this, a CCR2 (CCL2 and CCL7 shared receptor) antagonist PF-6309 was integrated into the PGEM micelle system. This dual PGEM/PF formulation effectively reduces tumor burden and induces anti-tumor immunity by reversing MDSC-mediated immunosuppression (Figure 3).

Mai et al. developed platelet membrane (PM) nanocarriers co-encapsulating metformin (Met) and IR780 (PM-IR780-Met NPs). The PM enhances tumor accumulation and retention, with IR780 acting as a photodynamic therapy agent to generate ROS, while Met reduces tumor oxygen consumption to improve PDT and induce ICD. This approach reverses tumor hypoxia, obstructs MDSC-regulated immunosuppressive pathways, and promotes T cell recruitment, showing potential for primary tumor elimination and metastasis control [51].

Lin et al. [52] developed Janus silica nanoparticles (JSNPs) to target MDSCs and enhance PD-L1 therapy by reshaping the TME. These JSNPs have two functional sides: one pH-responsive side that releases the PI3K-γ inhibitor IPI549 in the acidic TME to suppress MDSC activity, and a glutathione-sensitive side that releases CXCL9 cDNA in GSH-rich tumor cells. This dual action reduces MDSC-mediated immunosuppression and promotes cytotoxic lymphocyte recruitment, enhancing tumor sensitivity to PD-1/PD-L1 immune checkpoint therapy. By remodeling the TME, this nanoparticle system significantly improved immune responses, leading to reduced primary tumor growth, lower recurrence rates, and regression of distant tumors. This approach highlights the potential of MDSC-targeted strategies to boost PD-1/PD-L1 immunotherapy efficacy.

### Nanomedicine for modulating TAMs in PDAC

Macrophages in the TME can be classified as either M1 or M2. M1 macrophages are characterized by their pro-inflammatory and anti-tumor properties [53], while M2 macrophages primarily exhibit anti-inflammatory and tumor-promoting functions [54,55]. In the pancreatic cancer microenvironment, TAMs predominantly adopt an immunosuppressive M2 phenotype, which contributes to tumor progression [56]. Research has shown that TAMs can enhance the survival, migration, and invasion of cancer cells through their interactions with pancreatic cancer cells [57]. TAMs secrete cytokines like IL-10 and TGF-β, which inhibit the activity of effector T cells and NK cells, facilitating immune escape [58]. Furthermore, TAMs promote angiogenesis and tissue remodeling, further supporting tumor survival and expansion.

TAMs can be reprogrammed from a tumor-promoting M2 phenotype to an anti-tumor M1 phenotype via nanomedicine strategy. For instance, Moharil et al. demonstrated that folate receptor-targeted nanoparticles (FA-PGEM/DOX) significantly improved the targeting of M2-type macrophages in pancreatic cancer, resulting in a reduction in their numbers, an improved immune microenvironment, and enhanced anti-tumor efficacy [59]. Inhibiting CSF-1R has been shown to substantially deplete TAMs and increase the CD8^+^/CD4^+^ T cell ratio in mouse models, demonstrating efficacy in patients with diffuse-type giant cell tumors [60]. In addition, CD40-targeted therapies have been used to reprogram macrophages, as anti-CD40 therapy not only promotes tumor cell death but also contributes to matrix degradation, highlighting its potential for modifying the TME [61]. Li et al. [62] developed a composite of ROS-responsive nanogels loaded with LY3200882, a TGF-β inhibitor, and regorafenib. This formulation increased CD8^+^ T cell infiltration and shifted macrophages from an M2 (immunosuppressive) to M1 (anti-tumor) phenotype, showing promise in inhibiting tumor growth and metastasis.

Gao et al. developed an *in situ* thermosensitive chitosan hydrogel containing lipid immunoregulatory factor 5 (IRF5) mRNA/CCL5 siRNA (LPR) nanoparticle complex (LPR@CHG) that reprograms an anti-tumor immune niche. The chitosan hydrogel exhibits thermosensitivity due to the interaction of glycerophosphate with the polar chains of chitosan, along with the modulation of hydrophobic interactions by the glycerol moieties. The hydrogel up-regulates IRF5 and down-regulates CCL5 secretion, which contributes to a significant increase in M1 phenotype macrophages, enhancing T-cell-mediated immunity and controlling tumor growth [63]. This platform offers a promising immunotherapy strategy for pancreatic cancer with reduced systemic toxicity.

Tong et al. synthesized a tumor pH-sensitive polymer with a hydrophobic GEM prodrug (SPN@Pro-Gem), which self-assembles into nanoparticles that shrink at the tumor site for deep drug delivery [64]. This polymer was constructed by incorporating *N,N*-dipentylethylamine (DnPEA) moieties and monomethoxylpoly(ethylene glycol) into a PAMAM dendrimer, where the number of DnPEA moieties determines the degree of pH responsiveness. The nanoparticles not only kill tumor cells but also modulate the TME by reducing macrophages and myeloid suppressor cells while enhancing PD-L1 expression. This approach improves cytotoxic T cell infiltration and boosts the efficacy of checkpoint inhibitors in PDAC, offering a promising strategy for chemo-immunotherapy.

## Conclusion and perspectives

The unique characteristics of the PDAC TME, including dense ECM, hypoxic conditions, and the presence of immunosuppressive cells such as TAMs, MDSCs, and Tregs, create significant barriers to effective treatment. Nanocarriers have demonstrated substantial potential in overcoming the ECM barrier in PDAC by optimizing their physicochemical properties, thereby improving drug delivery efficiency and promoting deeper tumor penetration. Furthermore, nanocarriers can co-deliver immune-modulatory agents to regulate immune cells within the TME, reshaping the immune landscape and boosting PDAC immunotherapy.

However, the delivery efficiency of nanocarriers to PDAC still needs significant improvement. Future research should focus on developing more precise targeting strategies to selectively deliver drugs to immune cells or tumor cells within the TME, thereby enhancing therapeutic outcomes. In addition, while nanomedicines hold promise in triggering anti-tumor immune responses, they may also induce negative feedback mechanisms. Therefore, strategies to finely tune the immune microenvironment and overcome immune resistance are essential.

Understanding the interactions between nanocarriers and the TME, particularly their immunogenicity and immunomodulatory effects, is crucial for designing safe and effective nanocarriers. Moving forward, research should prioritize overcoming the unique challenges posed by PDAC’s dense, fibrotic stroma and immunosuppressive environment, with the goal of creating more efficient and targeted therapies that can improve patient outcomes.

**Summary**

- The dense extracellular matrix, immunosuppressive cells, and hypoxic conditions in pancreatic ductal adenocarcinoma (PDAC) create significant barriers to drug delivery and immune cell infiltration, contributing to therapy resistance.
- Nanocarriers offer a powerful tool to overcome these barriers by enhancing drug penetration, modulating immune cell function, and reprogramming the tumor microenvironment.
- Strategies targeting tumor-infiltrating T cells, natural killer cells, dendritic cells, myeloid-derived suppressor cells, and tumor-associated macrophages have shown promise in restoring anti-tumor immunity and improving therapeutic outcomes.
- Future research should focus on developing precise targeting strategies and addressing potential negative feedback mechanisms to enhance treatment effectiveness in PDAC.

## Supplementary material

online supplementary figure

## Abbreviations

APCs: antigen-presenting cells
DCs: dendritic cells
DOX: doxorubicin
ECM: extracellular matrix
GEM: gemcitabine
HA: hyaluronic acid
ICB: immune checkpoint blockade
ICD: immunogenic cell death
Lipo-NPs: liposome nanoparticles
MDSCs: myeloid-derived suppressor cells
MOF: metal-organic framework
NK: natural killer
NPs: nanoparticles
PDAC: pancreatic cancer
PGE2: prostaglandin E2
PGEM: gemcitabine-conjugated polymer
PM: platelet membrane
PM: platelet membrane
PSCs: pancreatic stellate cells
PTX: paclitaxel
ROS: reactive oxygen species
TAMs: tumor-associated macrophages
TLR: Toll-like receptor
TME: tumor microenvironment
Treg: regulatory T
VEGF: vascular endothelial growth factor
tdLNs: tumor-draining lymph nodes
