# Supplementary material for: Modulating immune cells within pancreatic ductal adenocarcinoma via nanomedicine
Source: Essays Biochem. 2025 May 26:EBC20243001. doi: 10.1042/EBC20243001 (PMC12224895; doi:10.1042/EBC20243001)
Supplement: online supplementary figure [file EBC-EBC20243001-s001.docx]

**Modulating Immune Cells within Pancreatic Ductal Adenocarcinoma via Nanomedicine**

**Junyi Lin^1,2^,** **Jingjing Sun^1,2^**^*^

1. Department of Pharmaceutical Sciences, College of Pharmacy, University of Nebraska Medical Center, Omaha, NE, 68106, USA.
2. Fred & Pamela Buffett Cancer Center, University of Nebraska Medical Center, Omaha, NE 68106, USA

*** Correspondence:** Jingjing Sun (jsun@unmc.edu).

**Abstract**

Pancreatic ductal adenocarcinoma (PDAC) is an aggressive malignancy characterized by a dense extracellular matrix (ECM) and a uniquely immunosuppressive tumor microenvironment (TME), which together form a formidable barrier that hinders deep drug penetration, limiting the efficacy of conventional therapies and leading to poor patient outcomes. Nanocarrier technology emerges as a promising strategy to improve treatment efficacy in PDAC. Nanocarriers can not only improve drug penetration through their adjustable physicochemical properties, but also effectively regulate immune cell function in pancreatic cancer TME and promote anti-tumor immune response. This mini-review discusses the effects of nanocarriers on the immune microenvironment of PDAC, analyzing their mechanisms in modulating immune cells, overcoming ECM barriers, and reshaping the TME.

**Introduction**

Pancreatic ductal adenocarcinoma (PDAC) is one of the most aggressive malignancies worldwide, characterized by late diagnosis, rapid progression, and resistance to conventional therapies(1-5). In the United States, PDAC remains a major health burden, with a five-year survival rate of just 11%(6). In Asia and Europe, the incidence rates are increasing. This situation has spurred extensive research efforts on understanding the role of the tumor microenvironment (TME) in PDAC progression and developing innovative therapeutic strategies. A significant challenge in treating PDAC lies in its complex TME, which consists of a dense extracellular matrix (ECM) and extensive desmoplasia (overproduction of fibrotic tissue due to excessive stromal activation)(7, 8). This not only restricts drug delivery, but also physically limits immune cell infiltration, leading to a highly immunosuppressive TME. In addition, various cytokines and chemokines are secreted in the TME, which promotes immune evasion, making PDAC particularly resistant to immunotherapies compared to other tumor types. Understanding and overcoming the barriers posed by the PDAC TME has therefore become a critical area of research.

The role of immune cells within the TME, such as tumor-infiltrating T cells, natural killer (NK) cells, dendritic cells (DCs) and tumor-associated macrophages (TAMs) is central to determining the outcome of the immune response. However, in PDAC, these immune cells often exhibit impaired function or are polarized toward immunosuppressive phenotypes due to the influence of cytokines, chemokines, and other soluble factors in the TME. Modulating these immune cells to restore their anti-tumor activity has emerged as a promising therapeutic strategy.

Nanomedicine has shown great potential in addressing the unique challenges of the PDAC TME. With their tunable size, surface properties, and ability to deliver therapeutic agents in a controlled and targeted manner, nanocarriers offer a powerful tool for enhancing drug delivery, modulating immune responses, and reprogramming immune cells within the TME. This mini-review explores recent research on using nanomedicine to modulate immune cells within the PDAC TME. We first discuss the key characteristics of the TME in PDAC and the challenges it poses for immunotherapy. Following this, we highlight recent advancements in targeting specific immune cell populations—such as tumor infiltrating T cells, NK cells, DCs, MDSC, and TAMs—using nanocarriers. By providing an overview of these emerging strategies, we aim to clarify why nanomedicine holds significant promise in the modulation of immune cells for the treatment of PDAC.

**Characteristics of pancreatic cancer TME**

PDAC presents a uniquely challenging TME compared to other solid tumors, largely due to its highly fibrotic and dense ECM. The fibrotic reaction, also known as desmoplasia, begins to form early around the lesions of pancreatic intraepithelial neoplasia (PanIN). In advanced stages, the ECM can account for up to 90% of the tumor volume. Pancreatic stellate cells (PSCs) are resident cells in the pancreas that play a crucial role in ECM production(9). Cytokines and growth factors, including insulin-like growth factor (IGF), TGF-β, and tumor necrosis factor-α (TNF-α), further stimulate PSC proliferation and exacerbate ECM synthesis, leading to abnormal fibrosis and ECM accumulation(10). The ECM of PDAC is rich in components such as collagen, proteoglycans, hyaluronic acid (HA), and fibronectin, forming a dense and impermeable barrier(11, 12). This fibrous matrix not only physically obstructs drug penetration and immune cell infiltration, but also increases interstitial pressure, reducing blood vessel density and causing low vascular permeability within the tumor(13).

In addition to creating physical barrier that restricts immune cell infiltration, components of the TME and tumor cells secrete various immunosuppressive molecules, such as IL-10, TGF-β, and vascular endothelial growth factor (VEGF). These factors promote the recruitment and maintenance of immunosuppressive cells such as TAMs, MDSCs, and Tregs, while simultaneously suppressing the activity of effector immune cells(**Fig 1)**. TGF-β, in particular, plays a central role in inhibiting NK cells and T cell activity, further reinforcing immunosuppression within the TME(14).

Due to the high interstitial pressure and poor vascularization resulting from the dense ECM, the PDAC TME is often hypoxic (low oxygen levels). Hypoxia leads to the stabilization of hypoxia-inducible factors (HIFs), which promote the expression of immunosuppressive cytokines and the recruitment of immunosuppressive cells. Tumor cells adapt to these hypoxic conditions by undergoing metabolic reprogramming, leading to increased nutrient competition between tumor cells and immune cells. This competition deprives anti-tumor immune cells, such as T cells, of essential nutrients, impairing their function and further enhancing immune evasion within the tumor.

Together, the dense ECM, activated PSCs, immunosuppressive factors, and hypoxic environment create a formidable barrier in PDAC, limiting the efficacy of conventional therapies and immunotherapies. Development of new strategies to overcome these barriers is critical for improving outcomes in PDAC.


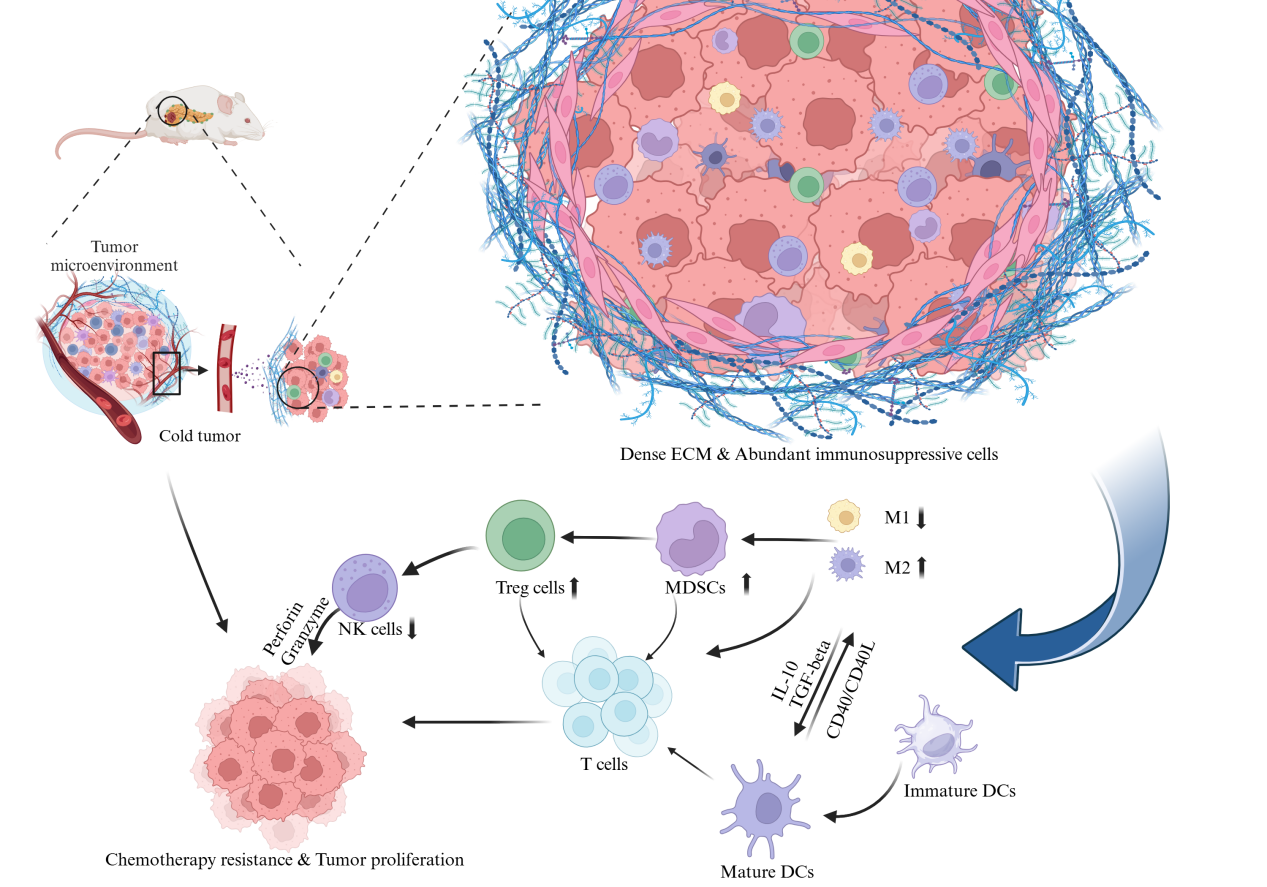


**Figure 1. Schematic representation of the uniquely challenging TME characteristic of PDAC.** The ECM and abundant immunosuppressive cells, such as Tregs and MDSCs, hinder immune infiltration and antitumor activity. Immune cells are dysregulated, with increased MDSCs, Tregs, and M2 macrophages, while cytotoxic T cells and NK cells are suppressed. These features promote chemotherapy resistance, tumor proliferation, and immune evasion, sustaining the cold tumor phenotype.

***Nanomedicine to Overcome the ECM Barrier***

Ultrasmall nanoparticles have been developed to effectively penetrate the stroma-rich environment of pancreatic tumors. Unlike larger particles, these smaller nanoparticles can better reach the tumor core, bypassing the dense ECM that typically restricts drug delivery(15). These nanocarriers can be used to deliver chemotherapeutics and immunotherapeutics deeply into the tumor core for better therapy. Nanoparticles can be designed to target stromal cells, such as cancer-associated fibroblasts, aim to reduce ECM production, ultimately decreasing the physical barrier to therapeutic agents(16). To overcome both ECM barrier and immunosuppressive microenvironment, Wang(17) et al. developed a small sized polyamino acid-based nanodrug incorporating the PSC activation inhibitor calcipotriol and anti-CXCL12 siRNA. This dual-delivery system works to penetrate pancreatic tumors, inactivate PSCs and downregulate CXCL12. This remodeled the TME by decreasing the ECM and immunosuppressive T cells, enhancing cytotoxic T cell infiltration and boosting the efficacy of immune checkpoint blockade (ICB) therapy in "cold" pancreatic tumors. Zhang(18) et al. designed a dual-warhead nanomedicine (TMV@LPS) by incorporating the hedgehog inhibitor (LDE225) into the bilayers of tumor cell membrane vesicles (TMV) and encapsulating PAMAM/SOX9 siRNA complexes to target the SOX9/CXCL5 axis. Utilizing the precision-guided stealth capability of the TMV coating, TMV@LPS enabled the targeted delivery of both the hedgehog inhibitor and siRNA, effectively reducing the secretion of collagen I, a key ECM component, and silencing the gene responsible for immune suppression. This approach successfully overcame both the ECM barrier and the immunosuppressive tumor microenvironment. Liu(19) et al. developed DOX-supported silica nanocarriers (DOX@HMSPHs) functionalized with hyaluronidase to degrade hyaluronic acid in the ECM, enhancing tumor penetration and inducing immunogenic cell death (ICD). The DOX@HMSPHs release DOX in acidic conditions, triggering ICD, promoting DC antigen presentation, and facilitating T cell activation.

***Nanomedicine Strategies to Modulate Tumor-Infiltrating T cells***

Tumor-infiltrating T cells are key immune components in the PDAC TME, comprising CD8^+^ effector T cells, CD4^+^ helper T cells and regulatory T cells (Tregs)(20). Among them, CD8^+^ T cells serve as primary cytotoxic agents against malignant tumor cells(21). However, PDAC is often classified as a “cold tumor”, characterized by limited T cell infiltration and functional exhaustion of T cells due to the highly immunosuppressive TME(22). In this hostile environment, factors such as tumor-secreted TGF-βand PD-L1 expression hinder the function of tumor infiltrating T cells, reducing their effectiveness against cancer cells(23, 24). CD8^+^ T cells, in particular, frequently exhibit an "exhausted" phenotype, marked by the overexpression of inhibitory receptors such as PD-1 and CTLA-4, which impairs their cytotoxic activity(25, 26).

To overcome these challenges, various nanomedicine strategies have been developed to modulate T cells and enhance their anti-tumor efficacy in PDAC. One promising approach by Zhu(27) et al. involves a TME-activable prodrug nanoparticle to enhance tumor penetration and achieve synergistic antitumor effects with chemotherapy and ICB. The nanoparticle contains a PD-L1 antagonist (DPPA) conjugated to a doxorubicin (DOX) prodrug, along with PEGylated DOX, which dissociates into small nanoparticles (<30 nm) in the TME, releasing DPPA. This co-delivery system improves tumor accumulation and penetration via transcytosis, directly killing tumor cells, promoting cytotoxic T cell infiltration, reducing Tregs, and inducing long-term immune memory to prevent recurrence and metastasis, shows promise for chemoimmunotherapy in solid tumors. This TME-activable nanoparticle offers a potential platform for enhancing immunochemotherapy in solid tumors. Another approach focuses on modulating the physical properties of nanoparticles to improve immune interactions. Yuan(28) et al. conducted an extensive study on liposome nanoparticles (Lipo-NPs) with varying elastic properties, focusing on their interactions with immune cells and their transport mechanisms from tumors to tumor-draining lymph nodes (tdLNs)**(Fig 2)**. They prepared Lipo-NPs with soft, moderate, and hard elasticity and observed distinct behaviors in immune cell interactions. Soft Lipo-NPs displayed an affinity for cell membranes, while moderate-elastic Lipo-NPs facilitated cargo delivery to macrophages through membrane fusion. In contrast, hard Lipo-NPs entered macrophages via a traditional cellular uptake pathway. Among the tested formulations, moderate-elastic Lipo-NPs loaded with the cGAMP agonist were particularly effective. These nanoparticles promoted significant tumor-infiltrating lymphocyte (TIL) infiltration by activating the interferon gene stimulator (STING) pathway and enhancing transport to tdLNs, which led to substantial anti-tumor effects and prolonged survival in a mouse melanoma model. This study highlights the potential synergies of moderately elastic Lipo-NPs with ICB therapy in preventing tumor immune escape. These findings offer valuable insights for developing immune-targeted delivery systems, especially in designing tdLN-targeted vaccines aimed at eradicating metastasis within tdLNs. In addition to these approaches, combining Toll-like receptor (TLR) 7/8 ligands with radiotherapy has shown promising results. Ye(29) et al. developed a linker-based strategy to control the activation of TLR7/8 agonists in PPS nanoparticles (PPS NPs). By attaching agonists to PPS NPs using different linkers, they enhanced therapeutic effects while reducing systemic toxicity. The alkyl linker selectively prolonged dendritic cell activation, limiting excessive immune responses and minimizing toxicity. This approach demonstrated strong anti-tumor effects and increased tumor-specific CD8^+^ T cells.

Since Tregs contribute to the immunosuppressive environment in PDAC, reducing their presence has been a focus of several nanomedicine strategies(30-33). For instance, Sun(34) et al. developed gemcitabine (GEM) conjugated polymers (PGEM) that co-deliver the indoleamine 2,3-dioxygenase 1 (IDO1) inhibitor NLG919 and chemotherapy agent paclitaxel (PTX). This combination reduced Tregs and increased CD4^+^ and CD8^+^ T cells expressing IFN-γ, enhancing the anti-tumor immune response and inhibiting tumor growth in PDAC models. This co-delivery strategy via nanoparticles offers an effective method for enhancing tumor penetration and improving immunochemotherapy outcomes.


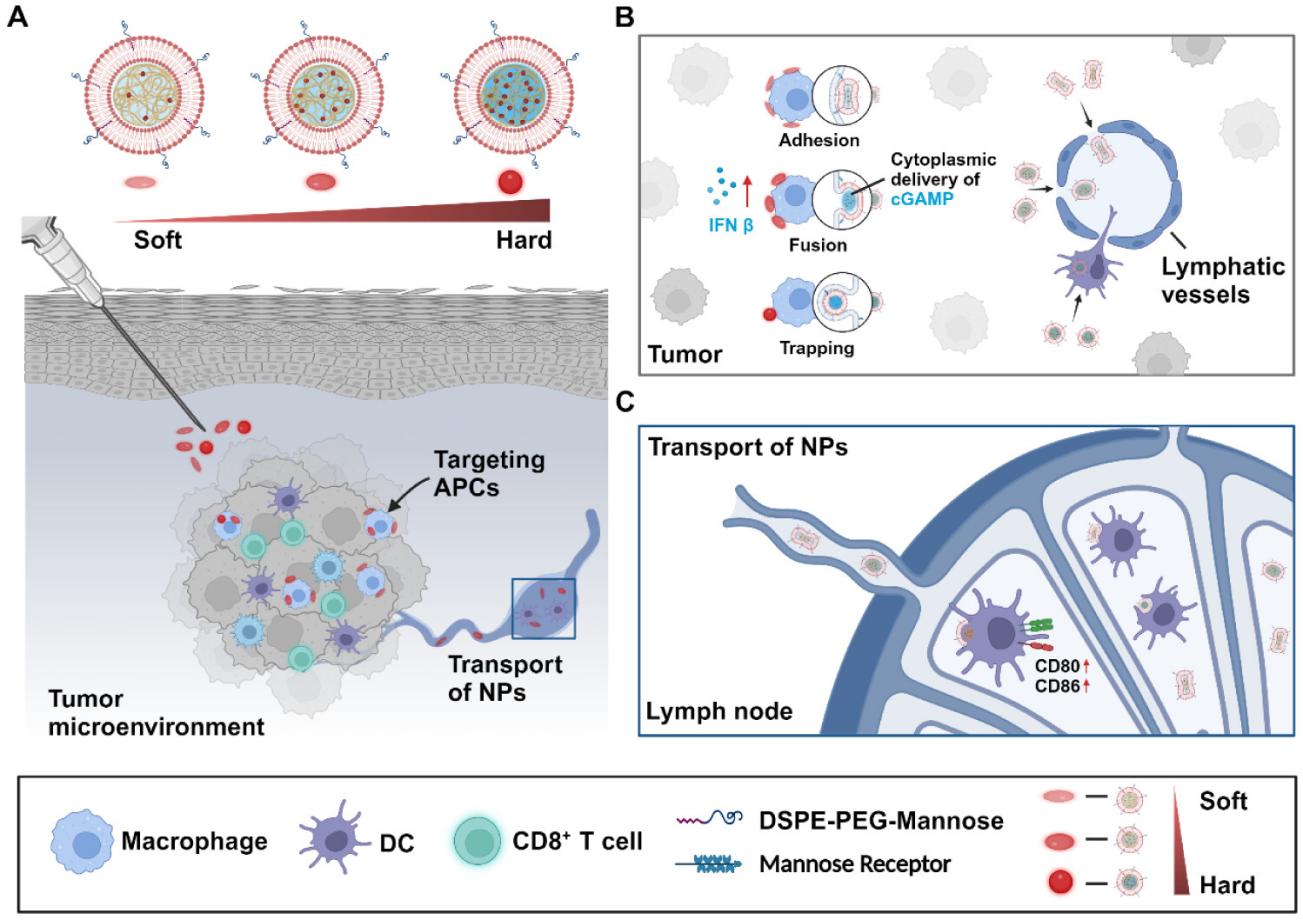


## Figure 2. Schematic representation of how the elasticity of Lipo-NPs influences core delivery and immune activation. (A) Depiction of local tumor administration for three types of Lipo-NPs. (B) Comparison of APC internalization and lymphatic vessel trafficking among the three Lipo-NPs with varying elastic properties. (C) The elasticity-dependent transport of Lipo-NPs to tumor-draining lymph nodes (tdLNs) following local tumor administration. Adapted with permission.(28) Copyright 2024, ACS Nano.

***Restoring NK Cells Function in PDAC through Nanomedicine***

NK cells are essential effector cells within the innate immune system, playing a crucial role in antitumor and antiviral responses. NK cells can recognize and directly eliminate tumor cells without prior activation. However, the functionality of NK cells is often impaired in tumors like pancreatic cancer due to immune suppressive factors and cellular interactions within the TME, which diminish their cytotoxic capacity, allowing tumor cells to evade immune surveillance(35).

Li(36) et al. designed a liposome conjugated with the tumor-homing peptide iRGD(c(CRGDKGPDC)), enabling enhanced tumor penetration and cell uptake for co-delivery of STING agonists and STAT3 inhibitors. These nanoparticles regulate the STING/STAT3 signaling axis and effectively inhibit tumor proliferation and survival. Treatment of the nanoparticles significantly increased the activation of NK cells and CD8^+^ T cells in tumors, resulting in robust innate and adaptive immune responses. Deng(37) et al. developed engineered nanogels coated with Panc02 cell membranes to inhibit tumor-derived prostaglandin E2 (PGE2) and enhance NK cell activation, promoting non-antigen-specific tumor elimination. The nanogels release acetaminophen on demand to reduce PGE2 secretion, while activated NK cells recruit immature DCs and stimulate their maturation, leading to antigen-specific CD8^+^ T cell proliferation. The nanogels demonstrated significant therapeutic effects against Panc02 pancreatic tumor growth and recurrence, particularly when combined with PD-L1 checkpoint blockade therapy, offering a novel strategy to improve immunotherapy in low-immunogenic tumors.

Yang(38) et al. demonstrate how a programmed nanoremodeler (DAS@P/H/pp) restores NK cell function in PDAC. Under the acidic tumor microenvironment, the nanocarrier undergoes charge reversal, triggering the release of hyaluronidase (HAase), which degrades the extracellular matrix. This process enhances the recruitment and infiltration of NK cells into deep tumor tissues, while promoting the delivery of immunoregulatory molecules and chemotherapy drugs. In a mouse model of pancreatic cancer, this nanomediated strategy significantly boosted the tumor-killing capabilities of NK92 cells. Near-infrared-II fluorescence imaging was used to monitor the treatment efficacy in real time. This approach highlights the potential of nanomedicine in overcoming the immunosuppressive microenvironment of PDAC and restoring NK cell anti-tumor function.

***Nanomedicine Based Restoration of Dendritic Cells Function***

DCs are essential components of antigen-presenting cells (APCs) that play a pivotal role in the TME of PDAC(31). Mature DCs present tumor antigens to CD8^+^ cytotoxic T cells via MHC-I and to CD4^+^ helper T cells via MHC-II, inducing a specific anti-tumor immune response(39). However, their function is often compromised in the highly immunosuppressive TME of PDAC(32, 33). Factors such as TGF-β, IL-10, and VEGF, released by tumor cells, inhibit DC maturation, impairing their antigen-presenting capability(40-43). These immature DCs lack the ability to effectively activate T cells and may instead promote immune evasion and tumor progression(44).

Recent studies have shown that metal-based nanoparticles (NPs), such as manganese (Mn) ions, can stimulate innate immunity both in vitro and ex vivo(45). Manganese-based metal-organic framework (MOF) NPs have been observed to accumulate specifically at tumor sites, where they function as reservoirs to release Mn ions locally. This targeted release enhances antitumor immunity by activating DCs while minimizing the toxicity commonly associated with traditional Mn sources(46). In a study with orthotopic pancreatic cancer-bearing mice, intravenous administration of PEGylated Mn-based MOF NPs over a two-week period increased the population of DCs and promoted their maturation, as indicated by elevated levels of costimulatory markers CD80, CD83, and CD86 in DC2.4 cells, alongside an increased percentage of CD8^+^ T cells within the tumor tissue(46).

To further boost DC function, Lorkowski (47) et al. developed a highly potent immune-stimulating nanoparticle (immuno-NP) carrying dual agonists targeting STING and TLR4 pathways. The study demonstrates that adjusting the ratio of two agonists in immune NPs achieves functional synergy, resulting in an 11-fold increase in IFNβ production compared to single-agonist variants. In an in-situ Panc02 mouse model of PDAC, systemic administration allowed immune NPs to localize predominantly in the APC-rich perivascular area of the tumor, where over 56% of dendritic cells absorbed the NPs. This led to a significant expansion of APCs, with an 11.5-fold increase in lymphocyte infiltration in dendritic cells and throughout pancreatic tumors compared to untreated controls.

***Targeting Myeloid-Derived Suppressor Cells (MDSC) in PDAC with Nanomedicine***

MDSCs are a heterogeneous population of bone marrow-derived cells that exhibit potent immunosuppressive functions within the TME. In pancreatic cancer patients, MDSC numbers are significantly elevated, and their presence is strongly associated with tumor progression and resistance to therapy(48). MDSCs inhibit the activity of both T cells and NK cell activity through the production of molecules such as nitric oxide synthase, arginase, and reactive oxygen species (ROS)(49). Gu(41) et al. et al. demonstrated that MDSCs in pancreatic cancer promote the production of ROS, leading to T cell apoptosis and functional impairment. In addition, MDSCs promote Treg recruitment and release IL-10 and TGF-β, further contributing to the immunosuppressive environment(42).

Various nanomedicine strategies target MDSCs to improve therapeutic outcomes. For example, LY364947, a TGF-β inhibitor, encapsulated in mesoporous silica nanoparticles, reduced MDSC-induced immunosuppression, enhancing gemcitabine delivery and decreasing tumor growth in pancreatic cancer models(43).

Lu(50) et al. introduced low molecular weight heparin-based nanoparticles (PLT/PTX NPs) that inhibited MDSC recruitment by blocking P-selectin/PSGL-1 interactions, thereby enhancing the immune microenvironment and reducing spontaneous metastasis in pancreatic cancer.

Wan(51)et al. developed a gemcitabine-conjugated polymer (PGEM) micelle system that shows excellent permeability in pancreatic tumor models and acts as a "STING agonist." PGEM activates STING signaling in both tumor cells and dendritic cells, enhancing NK and T cell responses. However, in tumor cells, PGEM-induced STING activation also promotes chemokines CCL2 and CCL7, recruiting immunosuppressive TAMs and MDSCs. To counter this, a CCR2 (CCL2 and CCL7 shared receptor) antagonist PF-6309 was integrated into the PGEM micelle system. This dual PGEM/PF formulation effectively reduces tumor burden and induces anti-tumor immunity by reversing MDSC-mediated immunosuppression**(Fig 3)**.

Mai et al. developed platelet membrane (PM) nanocarriers co-encapsulating metformin (Met) and IR780 (PM-IR780-Met NPs). The PM enhances tumor accumulation and retention, with IR780 acting as a photodynamic therapy agent to generate ROS, while Met reduces tumor oxygen consumption to improve PDT and induce ICD. This approach reverses tumor hypoxia, obstructs MDSC-regulated immunosuppressive pathways, and promotes T cell recruitment, showing potential for primary tumor elimination and metastasis control(52).

Lin(53) et al. developed Janus silica nanoparticles (JSNPs) to target MDSCs and enhance PD-L1 therapy by reshaping the TME. These JSNPs have two functional sides: one pH-responsive side that releases the PI3K-γ inhibitor IPI549 in the acidic TME to suppress MDSC activity, and a glutathione-sensitive side that releases CXCL9 cDNA in GSH-rich tumor cells. This dual action reduces MDSC-mediated immunosuppression and promotes cytotoxic lymphocyte recruitment, enhancing tumor sensitivity to PD-1/PD-L1 immune checkpoint therapy. By remodeling the TME, this nanoparticle system significantly improved immune responses, leading to reduced primary tumor growth, lower recurrence rates, and regression of distant tumors. This approach highlights the potential of MDSC-targeted strategies to boost PD-1/PD-L1 immunotherapy efficacy.


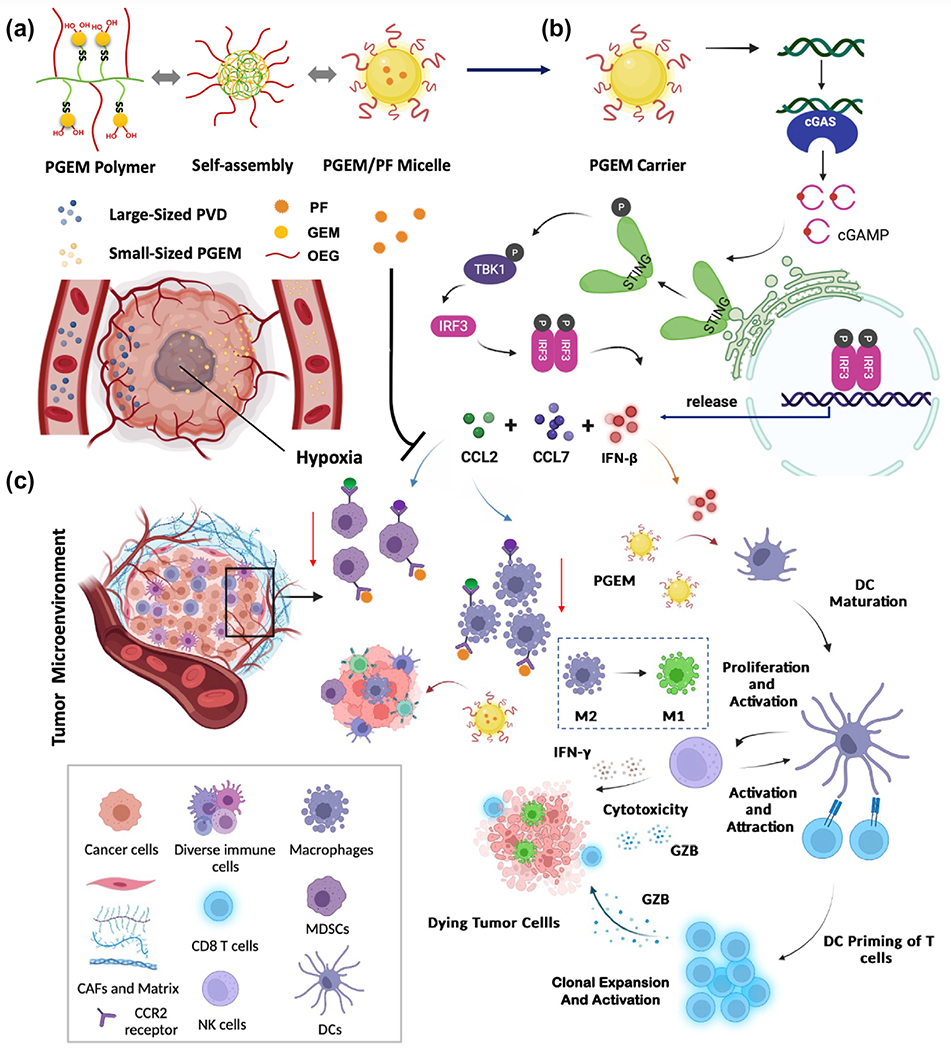


## Fig 3. PGEM/PF micelles demonstrate strong anti-tumor effects by reshaping the TME and enhancing STING pathway activation. (a) Small-sized PGEM/PF micelles were prepared to facilitate efficient penetration into PDAC solid tumors following systemic administration. (b) PGEM effectively activated the STING pathway, leading to the phosphorylation of TBK1 and IRF3, which in turn promoted the production of IFNβ, as well as chemokines CCL2 and CCL7. (c) The elevated IFNβ levels induced by PGEM enhanced anti-tumor immunity by facilitating dendritic cell (DC) maturation and stimulating both innate (NK cells) and adaptive (CD8 T cells) immune responses. Simultaneously, CCL2 and CCL7 contributed to recruiting tumor-associated macrophages (TAMs) and myeloid-derived suppressor cells (MDSCs) via CCR2 signaling. Additionally, PF released from PGEM/PF micelles further bolstered anti-tumor immunity by mitigating the immunosuppressive TME through its interplay with the STING pathway. Adapted with permission.(51) Copyright 2023, Mater Today.

***Nanomedicine for Modulating Tumor-Associated Macrophages (TAMs) in PDAC***

Macrophages in the TME can be classified as either M1 or M2. M1 macrophages are characterized by their pro-inflammatory and anti-tumor properties(54), while M2 macrophages primarily exhibit anti-inflammatory and tumor-promoting functions(55, 56). In the pancreatic cancer microenvironment, TAMs predominantly adopt an immunosuppressive M2 phenotype, which contributes to tumor progression(57). Research has shown that TAMs can enhance the survival, migration, and invasion of cancer cells through their interactions with pancreatic cancer cells(58). TAMs secrete cytokines like IL-10 and TGF-β, which inhibit the activity of effector T cells and NK cells, facilitating immune escape(59). Furthermore, TAMs promote angiogenesis and tissue remodeling, further supporting tumor survival and expansion.

TAMs can be reprogrammed from a tumor-promoting M2 phenotype to an anti-tumor M1 phenotype via nanomedicine strategy. For instance, Moharil et al. demonstrated that folate receptor-targeted nanoparticles (FA-PGEM/DOX) significantly improved the targeting of M2-type macrophages in pancreatic cancer, resulting in a reduction in their numbers, an improved immune microenvironment, and enhanced anti-tumor efficacy(60). Inhibiting CSF-1R has been shown to substantially deplete TAMs and increase the CD8^+^/CD4^+^ T cell ratio in mouse models, demonstrating efficacy in patients with diffuse-type giant cell tumors(61). In addition, CD40-targeted therapies have been used to reprogram macrophages, as anti-CD40 therapy not only promotes tumor cell death but also contributes to matrix degradation, highlighting its potential for modifying the TME(62). Li(63) et al. developed a composite of ROS-responsive nanogels loaded with LY3200882, a TGF-β inhibitor, and regorafenib. This formulation increased CD8^+^ T cell infiltration and shifted macrophages from an M2 (immunosuppressive) to M1 (anti-tumor) phenotype, showing promise in inhibiting tumor growth and metastasis.

Gao et al. developed an in situ thermosensitive chitosan hydrogel containing lipid immunoregulatory factor 5 (IRF5) mRNA/CCL5 siRNA (LPR) nanoparticle complex (LPR@CHG) that reprograms an anti-tumor immune niche. The chitosan hydrogel exhibits thermosensitivity due to the interaction of glycerophosphate with the polar chains of chitosan, along with the modulation of hydrophobic interactions by the glycerol moieties. The hydrogel upregulates IRF5 and downregulates CCL5 secretion, which contributes to a significant increase in M1 phenotype macrophages, enhancing T-cell-mediated immunity and controlling tumor growth(64). This platform offers a promising immunotherapy strategy for pancreatic cancer with reduced systemic toxicity.

Tong et al. synthesized a tumor pH-sensitive polymer with a hydrophobic gemcitabine prodrug (SPN@Pro-Gem), which self-assembles into nanoparticles that shrink at the tumor site for deep drug delivery(65). This polymer was constructed by incorporating N,N-dipentylethylamine (DnPEA) moieties and monomethoxylpoly(ethylene glycol) into a PAMAM dendrimer, where the number of DnPEA moieties determines the degree of pH responsiveness.The nanoparticles not only kill tumor cells but also modulate the TME by reducing macrophages and myeloid suppressor cells while enhancing PD-L1 expression. This approach improves cytotoxic T cell infiltration and boosts the efficacy of checkpoint inhibitors in PDAC, offering a promising strategy for chemo-immunotherapy.

**Conclusion and perspectives**

PDAC remains one of the most challenging malignancies to treat due to its dense ECM, immunosuppressive tumor TME, and resistance to conventional therapies. The unique characteristics of the PDAC TME, including extensive fibrosis, hypoxic conditions, and the presence of immunosuppressive cells such as TAMs, MDSCs, and Tregs, create significant barriers to effective treatment. Nanocarriers have demonstrated substantial potential in overcoming the ECM barrier in PDAC by optimizing their physicochemical properties, thereby improving drug delivery efficiency and promoting deeper tumor penetration. Furthermore, nanocarriers can co-deliver immune-modulatory agents to regulate immune cells within the TME, reshaping the immune landscape and boosting PDAC immunotherapy.

However, the delivery efficiency of nanocarriers to PDAC still needs significant improvement. Future research should focus on developing more precise targeting strategies to selectively deliver drugs to immune cells or tumor cells within the TME, thereby enhancing therapeutic outcomes. In addition, while nanomedicines hold promise in triggering anti-tumor immune responses, they may also induce negative feedback mechanisms. Therefore, strategies to finely tune the immune microenvironment and overcome immune resistance are essential.

Understanding the interactions between nanocarriers and the TME, particularly their immunogenicity and immunomodulatory effects, is crucial for designing safe and effective nanocarriers. Moving forward, research should prioritize overcoming the unique challenges posed by PDAC’s dense, fibrotic stroma and immunosuppressive environment, with the goal of creating more efficient and targeted therapies that can improve patient outcomes.

**Summary**

- The dense ECM, immunosuppressive cells, and hypoxic conditions in PDAC create significant barriers to drug delivery and immune cell infiltration, contributing to therapy resistance.
- Nanocarriers offer a powerful tool to overcome these barriers by enhancing drug penetration, modulating immune cell function, and reprogramming the TME.
- Strategies targeting tumor-infiltrating T cells, NK cells, DCs, MDSCs, and TAMs have shown promise in restoring anti-tumor immunity and improving therapeutic outcomes.
- Future research should focus on developing precise targeting strategies and addressing potential negative feedback mechanisms to enhance treatment effectiveness in PDAC.

**Competing Interests**

The authors declare that there are no competing interests associated with the manuscript.

**Funding**

This work was supported by National Institute of Health grants R01CA287091, P20GM121316 and Buffett Cancer Center Support Grant (CA036727).

**Author Contribution**

Both authors contributed with reviewing the literature and writing the manuscript.

**Abbreviations**

| Pancreatic cancer | PDAC | Doxorubicin | DOX |
| --- | --- | --- | --- |
| Extracellular matrix | ECM | Liposome nanoparticles | Lipo-NPs |
| Tumor microenvironment | TME | Tumor-draining lymph nodes | tdLNs |
| Natural killer | NK | Tumor-infiltrating lymphocytes | TILs |
| Dendritic cells | DCs | Toll-like receptor | TLR |
| Tumor-associated macrophages | TAMs | Gemcitabine | GEM |
| Pancreatic intraepithelial neoplasia | PanIN | Paclitaxel | PTX |
| Pancreatic stellate cells | PSCs | Prostaglandin E2 | PGE2 |
| Insulin-like growth factor | IGF | Antigen-presenting cells | APCs |
| Tumor necrosis factor-α | TNF-α | Nanoparticles | NPs |
| Hyaluronic acid | HA | Metal-organic framework | MOF |
| Vascular endothelial growth factor | VEGF | Myeloid-derived suppressor cells | MDSC |
| Hypoxia-inducible factors | HIFs | Gemcitabine-conjugated polymer | PGEM |
| Immunogenic cell death | ICD | Platelet membrane | PM |
| Regulatory T | Treg | Tumor-Associated Macrophages | TAMs |

**References**

1. Klein AP. Pancreatic cancer epidemiology: understanding the role of lifestyle and inherited risk factors. Nature reviews Gastroenterology & hepatology. 2021;18(7):493-502.

2. Vincent A, Herman J, Schulick R, Hruban RH, Goggins M. Pancreatic cancer. Lancet (London, England). 2011;378(9791):607-20.

3. Park W, Chawla A, O'Reilly EM. Pancreatic Cancer: A Review. Jama. 2021;326(9):851-62.

4. Stoffel EM, Brand RE, Goggins M. Pancreatic Cancer: Changing Epidemiology and New Approaches to Risk Assessment, Early Detection, and Prevention. Gastroenterology. 2023;164(5):752-65.

5. Ilic M, Ilic I. Epidemiology of pancreatic cancer. World journal of gastroenterology. 2016;22(44):9694-705.

6. Huang B, Huang H, Zhang S, Zhang D, Shi Q, Liu J, et al. Artificial intelligence in pancreatic cancer. Theranostics. 2022;12(16):6931-54.

7. Chan-Seng-Yue M, Kim JC, Wilson GW, Ng K, Figueroa EF, O'Kane GM, et al. Transcription phenotypes of pancreatic cancer are driven by genomic events during tumor evolution. Nature genetics. 2020;52(2):231-40.

8. Hosein AN, Brekken RA, Maitra A. Pancreatic cancer stroma: an update on therapeutic targeting strategies. Nature reviews Gastroenterology & hepatology. 2020;17(8):487-505.

9. Bazzichetto C, Conciatori F, Falcone I, Ciuffreda L. Translational Landscape of mTOR Signaling in Integrating Cues Between Cancer and Tumor Microenvironment. Advances in experimental medicine and biology. 2020;1223:69-80.

10. Pan-cancer analysis of whole genomes. Nature. 2020;578(7793):82-93.

11. Jacobetz MA, Chan DS, Neesse A, Bapiro TE, Cook N, Frese KK, et al. Hyaluronan impairs vascular function and drug delivery in a mouse model of pancreatic cancer. Gut. 2013;62(1):112-20.

12. Provenzano PP, Cuevas C, Chang AE, Goel VK, Von Hoff DD, Hingorani SR. Enzymatic targeting of the stroma ablates physical barriers to treatment of pancreatic ductal adenocarcinoma. Cancer cell. 2012;21(3):418-29.

13. Stopa KB, Kusiak AA, Szopa MD, Ferdek PE, Jakubowska MA. Pancreatic Cancer and Its Microenvironment-Recent Advances and Current Controversies. International journal of molecular sciences. 2020;21(9).

14. Liu W, Wei X, Li L, Wu X, Yan J, Yang H, et al. CCR4 mediated chemotaxis of regulatory T cells suppress the activation of T cells and NK cells via TGF-β pathway in human non-small cell lung cancer. Biochemical and biophysical research communications. 2017;488(1):196-203.

15. Chen H, Guo Q, Chu Y, Li C, Zhang Y, Liu P, et al. Smart hypoxia-responsive transformable and charge-reversible nanoparticles for the deep penetration and tumor microenvironment modulation of pancreatic cancer. Biomaterials. 2022;287:121599.

16. Patra JK, Das G, Fraceto LF, Campos EVR, Rodriguez-Torres MDP, Acosta-Torres LS, et al. Nano based drug delivery systems: recent developments and future prospects. Journal of nanobiotechnology. 2018;16(1):71.

17. Wang R, Hong K, Zhang Q, Cao J, Huang T, Xiao Z, et al. A nanodrug simultaneously inhibits pancreatic stellate cell activation and regulatory T cell infiltration to promote the immunotherapy of pancreatic cancer. Acta biomaterialia. 2023;169:451-63.

18. Zhang D, Song Q, Wang W, Li Q, Zhao Z, Jiang Y, et al. Unleashing a Dual‐Warhead Nanomedicine to Precisely Sensitize Immunotherapy for Pancreatic Ductal Adenocarcinoma. 2024;34(30):2315447.

19. Liu Q, Sun Y, Yin X, Li J, Xie J, Xie M, et al. Hyaluronidase-Functionalized Silica Nanocarrier for Enhanced Chemo-Immunotherapy through Inducing Immunogenic Cell Death. ACS applied bio materials. 2020;3(5):3378-89.

20. Han J, Khatwani N, Searles TG, Turk MJ, Angeles CV. Memory CD8(+) T cell responses to cancer. Seminars in immunology. 2020;49:101435.

21. Miller BC, Sen DR, Al Abosy R, Bi K, Virkud YV, LaFleur MW, et al. Subsets of exhausted CD8(+) T cells differentially mediate tumor control and respond to checkpoint blockade. Nature immunology. 2019;20(3):326-36.

22. Ou L, Liu S, Wang H, Guo Y, Guan L, Shen L, et al. Patient-derived melanoma organoid models facilitate the assessment of immunotherapies. EBioMedicine. 2023;92:104614.

23. Niu M, Yi M, Wu Y, Lyu L, He Q, Yang R, et al. Synergistic efficacy of simultaneous anti-TGF-β/VEGF bispecific antibody and PD-1 blockade in cancer therapy. Journal of hematology & oncology. 2023;16(1):94.

24. Kamada T, Togashi Y, Tay C, Ha D, Sasaki A, Nakamura Y, et al. PD-1(+) regulatory T cells amplified by PD-1 blockade promote hyperprogression of cancer. Proceedings of the National Academy of Sciences of the United States of America. 2019;116(20):9999-10008.

25. McRitchie BR, Akkaya B. Exhaust the exhausters: Targeting regulatory T cells in the tumor microenvironment. Frontiers in immunology. 2022;13:940052.

26. Bufe S, Zimmermann A, Ravens S, Prinz I, Buitrago-Molina LE, Geffers R, et al. PD-1/CTLA-4 Blockade Leads to Expansion of CD8(+)PD-1(int) TILs and Results in Tumor Remission in Experimental Liver Cancer. Liver cancer. 2023;12(2):129-44.

27. Zhu X, Li C, Lu Y, Liu Y, Wan D, Zhu D, et al. Tumor microenvironment-activated therapeutic peptide-conjugated prodrug nanoparticles for enhanced tumor penetration and local T cell activation in the tumor microenvironment. Acta biomaterialia. 2021;119:337-48.

28. Yuan P, Yan X, Zong X, Li X, Yang C, Chen X, et al. Modulating Elasticity of Liposome for Enhanced Cancer Immunotherapy. ACS nano. 2024;18(34):23797-811.

29. Zhang Y, Li Y, Xu Z, Xu L, Wang Y, Li N, et al. PPS-TLR7/8 agonist nanoparticles equip robust anticancer immunity by selectively prolonged activation of dendritic cells. Biomaterials. 2025;316:123032.

30. Göschl L, Scheinecker C, Bonelli M. Treg cells in autoimmunity: from identification to Treg-based therapies. Seminars in immunopathology. 2019;41(3):301-14.

31. Fu C, Jiang A. Dendritic Cells and CD8 T Cell Immunity in Tumor Microenvironment. Frontiers in immunology. 2018;9:3059.

32. Mandula JK, Chang S, Mohamed E, Jimenez R, Sierra-Mondragon RA, Chang DC, et al. Ablation of the endoplasmic reticulum stress kinase PERK induces paraptosis and type I interferon to promote anti-tumor T cell responses. Cancer cell. 2022;40(10):1145-60.e9.

33. Hato L, Vizcay A, Eguren I, Pérez-Gracia JL, Rodríguez J, Gállego Pérez-Larraya J, et al. Dendritic Cells in Cancer Immunology and Immunotherapy. Cancers. 2024;16(5).

34. Sun J, Wan Z, Chen Y, Xu J, Luo Z, Parise RA, et al. Triple drugs co-delivered by a small gemcitabine-based carrier for pancreatic cancer immunochemotherapy. Acta biomaterialia. 2020;106:289-300.

35. Vivier E, Tomasello E, Baratin M, Walzer T, Ugolini S. Functions of natural killer cells. Nature immunology. 2008;9(5):503-10.

36. Li R, Liu R, Xu Y, Zhang S, Yang P, Zeng W, et al. Suppressing Pancreatic Cancer Survival and Immune Escape via Nanoparticle-Modulated STING/STAT3 Axis Regulation. Bioconjugate chemistry. 2024.

37. Deng J, Xu W, Lei S, Li W, Li Q, Li K, et al. Activated Natural Killer Cells-Dependent Dendritic Cells Recruitment and Maturation by Responsive Nanogels for Targeting Pancreatic Cancer Immunotherapy. Small (Weinheim an der Bergstrasse, Germany). 2022;18(44):e2203114.

38. Yang X, Li C, Yang H, Li T, Ling S, Zhang Y, et al. Programmed Remodeling of the Tumor Milieu to Enhance NK Cell Immunotherapy Combined with Chemotherapy for Pancreatic Cancer. Nano letters. 2024;24(11):3421-31.

39. Hsu DH, Paz P, Villaflor G, Rivas A, Mehta-Damani A, Angevin E, et al. Exosomes as a tumor vaccine: enhancing potency through direct loading of antigenic peptides. Journal of immunotherapy (Hagerstown, Md : 1997). 2003;26(5):440-50.

40. Wculek SK, Cueto FJ, Mujal AM, Melero I, Krummel MF, Sancho D. Dendritic cells in cancer immunology and immunotherapy. Nature reviews Immunology. 2020;20(1):7-24.

41. Gu H, Deng W, Zheng Z, Wu K, Sun F. CCL2 produced by pancreatic ductal adenocarcinoma is essential for the accumulation and activation of monocytic myeloid-derived suppressor cells. Immunity, inflammation and disease. 2021;9(4):1686-95.

42. Chen Q, Yin H, He J, Xie Y, Wang W, Xu H, et al. Tumor Microenvironment Responsive CD8(+) T Cells and Myeloid-Derived Suppressor Cells to Trigger CD73 Inhibitor AB680-Based Synergistic Therapy for Pancreatic Cancer. Advanced science (Weinheim, Baden-Wurttemberg, Germany). 2023;10(33):e2302498.

43. Meng H, Zhao Y, Dong J, Xue M, Lin YS, Ji Z, et al. Two-wave nanotherapy to target the stroma and optimize gemcitabine delivery to a human pancreatic cancer model in mice. ACS nano. 2013;7(11):10048-65.

44. Palucka K, Banchereau J. Cancer immunotherapy via dendritic cells. Nature reviews Cancer. 2012;12(4):265-77.

45. Peng P, Cao J, Cheng W, Ming H, He B, Duan X, et al. Manganese dioxide-based in situ vaccine boosts antitumor immunity via simultaneous activation of immunogenic cell death and the STING pathway. Acta biomaterialia. 2025.

46. Liu J, Yang L, Cao X, Chen M, Li J, Wang X, et al. PEGylated Mn containing MOF nanoparticles for potential immunotherapy of pancreatic cancer via manganese induced activation of anti-tumor immunity. 2021;42:100409.

47. Lorkowski ME, Atukorale PU, Bielecki PA, Tong KH, Covarrubias G, Zhang Y, et al. Immunostimulatory nanoparticle incorporating two immune agonists for the treatment of pancreatic tumors. Journal of controlled release : official journal of the Controlled Release Society. 2021;330:1095-105.

48. Gabrilovich DI, Nagaraj S. Myeloid-derived suppressor cells as regulators of the immune system. Nature reviews Immunology. 2009;9(3):162-74.

49. Youn JI, Nagaraj S, Collazo M, Gabrilovich DI. Subsets of myeloid-derived suppressor cells in tumor-bearing mice. Journal of immunology (Baltimore, Md : 1950). 2008;181(8):5791-802.

50. Lu Z, Long Y, Wang Y, Wang X, Xia C, Li M, et al. Phenylboronic acid modified nanoparticles simultaneously target pancreatic cancer and its metastasis and alleviate immunosuppression. European journal of pharmaceutics and biopharmaceutics : official journal of Arbeitsgemeinschaft fur Pharmazeutische Verfahrenstechnik eV. 2021;165:164-73.

51. Wan Z, Huang H, West RE, 3rd, Zhang M, Zhang B, Cai X, et al. Overcoming pancreatic cancer immune resistance by codelivery of CCR2 antagonist using a STING-activating gemcitabine-based nanocarrier. Materials today (Kidlington, England). 2023;62:33-50.

52. Mai X, Zhang Y, Fan H, Song W, Chang Y, Chen B, et al. Integration of immunogenic activation and immunosuppressive reversion using mitochondrial-respiration-inhibited platelet-mimicking nanoparticles. Biomaterials. 2020;232:119699.

53. Lin X, Li F, Guan J, Wang X, Yao C, Zeng Y, et al. Janus Silica Nanoparticle-Based Tumor Microenvironment Modulator for Restoring Tumor Sensitivity to Programmed Cell Death Ligand 1 Immune Checkpoint Blockade Therapy. ACS nano. 2023;17(15):14494-507.

54. Mosser DM, Edwards JP. Exploring the full spectrum of macrophage activation. Nature reviews Immunology. 2008;8(12):958-69.

55. Biswas SK, Mantovani A. Macrophage plasticity and interaction with lymphocyte subsets: cancer as a paradigm. Nature immunology. 2010;11(10):889-96.

56. Tian C, Clauser KR, Öhlund D, Rickelt S, Huang Y, Gupta M, et al. Proteomic analyses of ECM during pancreatic ductal adenocarcinoma progression reveal different contributions by tumor and stromal cells. Proceedings of the National Academy of Sciences of the United States of America. 2019;116(39):19609-18.

57. Weizman N, Krelin Y, Shabtay-Orbach A, Amit M, Binenbaum Y, Wong RJ, et al. Macrophages mediate gemcitabine resistance of pancreatic adenocarcinoma by upregulating cytidine deaminase. Oncogene. 2014;33(29):3812-9.

58. Mitchem JB, Brennan DJ, Knolhoff BL, Belt BA, Zhu Y, Sanford DE, et al. Targeting tumor-infiltrating macrophages decreases tumor-initiating cells, relieves immunosuppression, and improves chemotherapeutic responses. Cancer research. 2013;73(3):1128-41.

59. Liu S, Ren J, Ten Dijke P. Targeting TGFβ signal transduction for cancer therapy. Signal transduction and targeted therapy. 2021;6(1):8.

60. Moharil P, Wan Z, Pardeshi A, Li J, Huang H, Luo Z, et al. Engineering a folic acid-decorated ultrasmall gemcitabine nanocarrier for breast cancer therapy: Dual targeting of tumor cells and tumor-associated macrophages. Acta pharmaceutica Sinica B. 2022;12(3):1148-62.

61. Ries CH, Cannarile MA, Hoves S, Benz J, Wartha K, Runza V, et al. Targeting tumor-associated macrophages with anti-CSF-1R antibody reveals a strategy for cancer therapy. Cancer cell. 2014;25(6):846-59.

62. Beatty GL, Chiorean EG, Fishman MP, Saboury B, Teitelbaum UR, Sun W, et al. CD40 agonists alter tumor stroma and show efficacy against pancreatic carcinoma in mice and humans. Science (New York, NY). 2011;331(6024):1612-6.

63. Li Z, Xu W, Yang J, Wang J, Wang J, Zhu G, et al. A Tumor Microenvironments-Adapted Polypeptide Hydrogel/Nanogel Composite Boosts Antitumor Molecularly Targeted Inhibition and Immunoactivation. Advanced materials (Deerfield Beach, Fla). 2022;34(21):e2200449.

64. Gao C, Cheng K, Li Y, Gong R, Zhao X, Nie G, et al. Injectable Immunotherapeutic Hydrogel Containing RNA-Loaded Lipid Nanoparticles Reshapes Tumor Microenvironment for Pancreatic Cancer Therapy. Nano letters. 2022;22(22):8801-9.

65. Tong QS, Miao WM, Huang H, Luo JQ, Liu R, Huang YC, et al. A Tumor-Penetrating Nanomedicine Improves the Chemoimmunotherapy of Pancreatic Cancer. Small (Weinheim an der Bergstrasse, Germany). 2021;17(29):e2101208.
